# Effects of propofol and sevoflurane on T-cell immune function and Th cell differentiation in children with SMPP undergoing fibreoptic bronchoscopy

**DOI:** 10.1080/07853890.2022.2121416

**Published:** 2022-11-12

**Authors:** Hui Yu, Lin Chen, Cheng-Jin Yue, Heng Xu, Jing Cheng, Elyse M. Cornett, Alan D. Kaye, Ivan Urits, Omar Viswanath, Henry Liu

**Affiliations:** a Department of Anesthesiology, Hubei Women and Children’s Hospital, Tongji Medical College, Huazhong University Science & Technology, Wuhan, Hubei, China; b Departments of Anesthesiology and Pharmacology, Toxicology & Neuroscience, LSU Health Shreveport, Shreveport, LA, USA; c Southcoast Health, Southcoast Physicians Group Pain Medicine, Wareham, MA, USA; d University of Arizona College of Medicine-Phoenix, Phoenix, AZ, USA; e Department of Anesthesiology, Creighton University School of Medicine, Omaha, NE, USA; f Valley Anesthesiology and Pain Consultants – Envision Physician Services, Phoenix, AZ, USA; g Department of Anesthesiology & Perioperative Medicine, Milton S. Hershey Medical Center, Penn State College of Medicine, Hershey, PA, USA

**Keywords:** Propofol, sevoflurane, immune function, severe mycoplasmal pneumonia, fibreoptic bronchoscopy, bronchial lavage

## Abstract

**Objectives:**

The potentially different effects of commonly used anaesthetic agents propofol and sevoflurane on T-cell immune function and Th cell differentiation were investigated in patients with severe mycoplasmal pneumonia (SMPP) undergoing fibreoptic bronchoscopy.

**Methods:**

Sixty children (2–12 years of age) with SMPP were randomized into the sevoflurane group and the propofol group. Patients in the sevoflurane group were anaesthetised with inhalational sevoflurane and intravenous remifentanil. Patients in the propofol group were anaesthetised with intravenous propofol and remifentanil. Patients in both groups underwent fibreoptic bronchoscopy and lavage therapy. We compared the clinical outcomes, cellular immunity function, and Th cell differentiation into Th1 and Th2 levels in both groups.

**Results:**

There was no significant difference in clinical outcomes and hospital stay between the two groups (7.94 vs 7.36, *p* > .05). However, the CD3^+^ T cells, CD4^+^ T cells, and CD4^+^/CD8^+^ in the propofol group were significantly higher than those in the sevoflurane group (T1 51.96 vs 48.33, T2 58.08 vs 55.31, *p* < .05). The ratio of Th_1_/Th_2_ in the two groups was significantly increased postoperatively in both groups (Sevoflurane 8.53 vs 7.23, Propofol 9.35 vs 7.18), and the propofol group was significantly higher than the sevoflurane group (9.35 vs 8.53, *p* < .05).

**Conclusions:**

Propofol might have a less inhibitory effect on T lymphocytes in children with SMPP than sevoflurane. And propofol may have less impact on the differentiation of Th cells into Th_1_ cells and better preserving the Th_1_/Th_2_ ratio than sevoflurane.

**KEY MESSAGES**
- The pathogenesis of SMPP is still unclear, likely through alveolar infiltration with neutrophils and lymphocytes, lymphocyte/plasma cell infiltrates in the peri-bronchovascular area, and immune dysfunction.
- Recent experimental and clinical studies showed that sevoflurane might have immunosuppressive effects, and multiple studies confirmed that the immune function of children with SMPP had been reduced.
- This study found that propofol administered in children with SMPP had a less inhibitory effect on T lymphocytes than inhalational sevoflurane, had little inhibitory effect on the differentiation of Th cells into Th1 cells, and better preserve Th1/Th2 ratio and maintain the balanced immune function.

## Introduction

The incidence of severe mycoplasmal pneumoniae pneumonia (SMPP) has been steadily trending up in recent years. Although mycoplasma pneumonia (MP) is a benign self-limiting disease, SMPP can potentially cause severe damages to the lung structure and negatively affect pulmonary function [1]. Fibreoptic bronchoscopy and lavage are important diagnostic and therapeutic options for patients with SMPP. Fibreoptic bronchoscopy and alveolar lavage in children are generally performed under general anaesthesia [2]. Previous studies unveiled that anaesthetic drugs might have some T-cell immunosuppressive effects, and different anaesthetic agents might have different effects on T-cell immune function. Zhang et al. showed that when compared to propofol, sevoflurane exerted significantly more inhibitory effects on patients’ CD3+ and CD4+ percentages and more impact on CD4+/CD8+ ratio. Other studies also showed that the development of mycoplasmal pneumonia is closely related to the Th1/Th2 imbalance, and Th2 cells tend to predominate [3,4]. The immunosuppressive effects and the effects on Th1/Th2 balance of anaesthetic agents may affect the development and prognosis of children with SMPP. This study aimed to investigate the effects of propofol and sevoflurane on T-cell immune function and Th1/Th2 balance and the impact on clinical outcome in children with SMPP undergoing fibreoptic bronchoscopy and alveolar lavage.

## Methods

### Basic information

From March 2018 to March 2019, 60 children, 33 male, and 27 female children, aged 2 ∼ 12 years, diagnosed with SMPP, were admitted to our paediatric intensive care unit (PICU) at Hubei Women and Children’s Hospital. These children were randomly divided into the sevoflurane group and the propofol group, with 30 cases in each group. There were 16 males and 14 females with an average age of (7.21 ± 2.04) in the propofol group. There were 17 males and 13 females with an average age of (6.94 ± 1.98) in the sevoflurane group. There was no significant difference in demographic data between the two groups. This clinical study was approved by our hospital ethics committee, Hubei Women and Children’s Hospital Medical Ethnic Committee HBHS #2018-0138. Clinical trial number: ChiCTR1900022050.

### Inclusion and exclusion criteria

The inclusion criteria were: (Criteria 1) between the ages of 2 and 12 years; (Criteria 2) all children were examined at the time of admission and the pathogen culture met the diagnostic criteria for SMPP [5]. (Criteria 3) Persistent high fever (≧38.5 °C) with cough for 48 h or more; (Criteria 4) poor response to single macrolide antibiotics treatment with symptoms no improvement or even progressive exacerbation, and chest X-ray (CXR) showed consolidation of the lungs with atelectasis, or varying degrees of pleural effusion, serological MP-IgM positive and the CXR findings were consistent with mycoplasma lobar pneumonia; (Criteria 5) signed informed consent.

The exclusion criteria were: (Criteria 1) children with immunologic depression, liver and kidney dysfunction, cardiovascular and endocrine disorders; (Criteria 2) Children who were allergic to propofol or other anaesthetic agents; (Criteria 3) children who had anaesthesia and analgesics within three months; (Criteria 4) children who underwent fibreoptic bronchoscopy and immunomodulatory drug therapy within a month; (Criteria 5) Pathogen test of alveolar lavage fluid showed mixed infection of common bacteria or common respiratory viruses.

### Anaesthetic management

All children were transported to the operating room with established venous access but without preoperative medication. All patients had standard ASA monitoring, including ECG, MAP, HR, SpO_2_, End-tidal CO_2_, temperature. Patients in the propofol group received intravenous propofol 2 ∼ 4mg/kg and remifentanil 1 μg/kg for induction, and continuous intravenous infusion of propofol (9 ∼ 15mg/kg/*h*, maintaining BIS at 40–50) and remifentanil (1 μg/kg/min). Patients in the sevoflurane group received combined anaesthesia of inhalational 8% sevoflurane and intravenous remifentanil 1 μg/kg for induction, intraoperatively patients received 2 ∼ 5% sevoflurane inhalation and continuous infusion of remifentanil (1 μg/kg/min) to maintain BIS at 40 ∼ 50 level.

All patients had laryngeal mask airway (LMA) inserted after induction of general anaesthesia. Then mechanical ventilation was used during the procedure. The tidal volume was set at 8 ∼ 10 mL/kg, and the respiratory rate was dialled to maintain PETCO2 at 35 ∼ 45 mmHg. After an adequate depth of general anaesthesia and satisfactory ventilation through LMA was established, the fibreoptic bronchoscopy started by inserting the bronchoscope *via* LMA, and topical 2% lidocaine at the dose of 1 mg/kg is sprayed through the injection hole on the glottis, followed by fibreoptic bronchoscopy and bronchoalveolar lavage. At the end of the procedure, patients were emerged and extubated and transported to paediatric PICU.

### Sample collection and storage

A peripheral blood sample was used for all immunological tests. We collected 8 mL of peripheral venous blood at three time points: before induction of general anaesthesia (T0), end of anaesthesia (T1), and 24 h after the end of anaesthesia (T2). The anaesthesia time of propofol group was 30–62 min, with an average of 50.2 ± 1.8 min. The mean operation time was 35.1 ± 2.2 min (range from 20 min to 40 min); The anaesthesia time of sevoflurane group was 33–58 min, with an average of 49.9 ± 1.3 min. The mean operation time was 37.3 ± 1.4 min (range from 22 min to 45 min). There was no significant difference in the average anaesthesia time and operation time between the two groups.

All samples were stored at −80 °C. All samples were tested for immunological parameters together as one batch. Out of the 8 mL, 4 mL was used to detect the T-cell percentage of CD3+, CD4+, CD8+ cells in peripheral blood, and CD4+/CD8+ ratio in each sample by flow cytometry. The remaining 4 mL of blood was used for flow cytometry detection of Th1/Th2 ratio in the blood sample. There were three kinds of cell suspensions labelled with fluorescent antibodies of CD3, CD8 and IFN- γ and three kinds of cell suspensions labelled with fluorescent antibodies of CD3, CD8 and IL-4. Then the cell suspension was detected by flow cytometry, The percentage of Th1 cells labelled with fluorescent antibodies of CD3 + CD8-IFN-γ+ and the percentage of Th2 cells labelled with fluorescent antibodies of CD3 + CD8-IL-4+ were analysed by multi parameter analysis system of flow cytometry. The results of flow cytometry (the percentage of the number of cells combined with the fluorescent labelled antibody and the corresponding cell surface antigen and the total number of cells passing the flow cytometry) indicate the percentage (%) of the number of cell surface antigen positive cells in the total number of cells in the sample.

Heparin anticoagulated peripheral blood was isolated from peripheral blood mononuclear cells (PBMC) by density gradient centrifugation, and mononuclear cell layers (PBMCs) were treated with equal amounts of RPMI-1640 medium (10% calf serum, 100 U/mL penicillin, and 0.1). Mix ng/mL streptomycin) (Gibco), adjust the concentration of PBMCs to 2 × 10^6–8^/mL, and add stimulating hormone (Alexis Biochemicals, San Diego, CA, USA) phorbol myristate acetate (PMA) to the mixed suspension. Ionase and brefeldin (BFA) were incubated for 4 h at 37 °C in a 5% CO_2_ incubator. The cultured cells were added to phosphate-buffered saline (PBS buffer, Alexis Biochemicals, San Diego, CA), centrifuged at 1000 r/min for 5 min, and the supernatant was discarded. After centrifugation, the cells were each added with four tubes labelled with ABCD. Each tube was labelled with 4 μL of CD3-PE Cy5, CD8-PE Cy7 antibody (eBiochemicals, San Diego, CA, USA), incubated for 15 min in the dark, and reused. After PBS buffer was washed, a rupturing agent (FIX & PERM, e Bioscience) was added, and then the Th1 labelled antibody IFN-γ-FITC, the control antibody Goat Anti-Mouse IgG-FITC, and the Th2 labelled antibody IL-4-PE, the control antibody Goat Anti-Mouse IgG-PE, (eBiochemicals, San Diego, CA, USA) were respectively used in the ABCD tubes, incubated for 15 min in the dark. Each tube was incubated with PBS buffer and then detected by flow cytometry (Becton Dickerson, San Jose, USA). CD3 + CD8-IFN-γ+ was Th1 cells and CD3 + CD8-IL-4+ was Th2 cells. The Th1/Th2 cell ratio was recorded (The most important feature of flow cytometry for determining the percentage of Th1 cells and Th2 cells is the gating of helper T cells (CD4+ T lymphocytes). The experiment involves how to correctly select the problem of Th. The surface marker of Th cells is CD3 + CD4+, and when stimulated with stimulating hormone phorbol ester (PMA) for 4 h, it is found that CD4+ cells are significantly reduced or even disappeared because PMA induces endocytosis of cell-surface CD4 molecules. CD4 cells are considered to be CD3 + CD4+ cells by CD3 and CD8 [6,7].

### Observation parameters

The observation parameters of this study included:

- Immunological parameters.
- The overall clinical outcome at 7 days after the procedure was categorised as: A. Markedly effective: fever, cough and other clinical symptoms completely disappeared, no abnormalities found on chest X-ray examination; B. Effective: fever, cough and other clinical symptoms were relieved. Chest X-ray examination showed that the lung infiltration was reduced by more than 50%. C. Ineffective: the clinical symptoms of the child and the chest X-ray images did not change significantly.
- The length of hospital stays.

### Statistical processing

SPSS 19.0 software is used for data processing. The measurement data are expressed as mean ± standard deviation ($\bar{x}\pm s$). The count data (%) were tested using χ^2^, and *p* < .05 indicates a significant difference.

## Results

### Comparison of two groups of cellular immune indicators

There was no significant difference in cellular immune index between the two groups before induction of anaesthesia (*p* > .05). At the end of anaesthesia, CD3+, CD4+ and CD4+/CD8+ were lower than the corresponding indexes before induction (*p* < .05), sevoflurane. The group was lower than the propofol group, the difference was statistically significant (*p* < .05), but the cell immunity was significantly restored 24 h after the end of anaesthesia. The CD3+, CD4+ and CD4+/CD8+ in the propofol group were significantly higher than those in the sevoflurane group (*p* < .05), see Table 1. The CD3+, CD4+ and CD4+/CD8 at T1 were lower than those at T0 (*p* < .05); The CD3+, CD4+ and CD4+/CD8 in sevoflurane group were lower than those of propofol group at T1, and the difference was statistically significant (*p* < .05).

**Table 1.** Comparison of two groups of cellular immune indicators.

Group	CD3+ (%)			CD4+ (%)		
	T0	T1	T2	T0	T1	T2
Propofol group	54.37 ± 3.43	51.96 ± 3.70*	58.08 ± 3.98#	30.95 ± 1.96	27.94 ± 1.89*	33.06 ± 1.94#
Sevoflurane group	54.17 ± 3.67	48.33 ± 3.59*	55.31 ± 3.61#	30.32 ± 2.03	23.42 ± 1.86*	28.95 ± 1.98#
T	0.375	4.642	4.956	0.385	4.725	4.821
P	.758	.000	.000	.735	.000	.000

Group	CD8+ (%)			CD4+/CD8+		
	T0	T1	T2	T0	T1	T2
Propofol group	24.75 ± 1.98	24.73 ± 1.79	25.76 ± 1.94	1.21 ± 0.11	1.18 ± 0.08*	1.28 ± 0.13#
Sevoflurane group	24.76 ± 2.01	25.37 ± 1.85	26.04 ± 1.95	1.22 ± 0.10	0.96 ± 0.11*	1.16 ± 0.12#
T	0.351	1.671	1.296	0.354	3.991	3.821
P	.795	.102	.231	.785	.000	.000

*Indicates comparison.

With T0, *p < .05; # indicates comparison with T1, #*p* < .05.

### Comparison of Th1/Th2 ratios between the two groups

**Figure 1.**
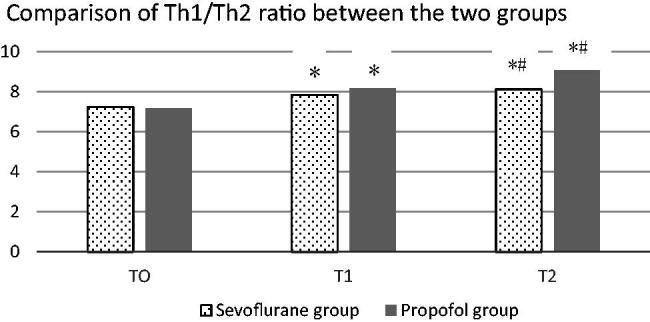
Comparison of Th1/Th2 ratios between the two groups: *Indicates comparison with t0, *p < .05; # indicates comparison with t1, #*p* < .05.

There was no significant difference in the ratio of Th1/Th2 between the two groups before the induction of anaesthesia (*p* > .05). At the end of anaesthesia, the ratio of Th1/Th2 in the two groups was slightly higher than that before the induction (*p* < .05). There was also no difference (*p* > .05). The ratio of Th1/Th2 was significantly increased in both groups 24 h after anaesthesia, and the ratio of Th1/Th2 in the propofol group was significantly higher than that in the control group (*p* > .05). See Table 2 and Figure 1. According to the cytokine spectrum of CD4 + Th cells, CD4 + Th cells were divided into two independent subpopulations: Th1 and Th2. Th1 cells mainly secreted IL-2, IL-12 and IFN- γ And TNF- β Th2 cells mainly secrete IL-4, IL-5, IL-6 and IL-10. Its main function is to stimulate B cell proliferation and produce antibodies, which is related to humoral immunity. More and more evidence shows that the imbalance of Th1/Th2 ratio is a key link in the regulation of immune response. The imbalance of Th1/Th2 ratio can lead to diseases, such as chronic infection, autoimmune diseases or allergic reactions. Some studies have shown that the development of mycoplasma pneumonia is closely related to Th1/Th2 imbalance, and Th2 cells tend to be dominant.

**Table 2.** Comparison of Th1/Th2 ratios between the two groups.

Group	T0	T1	T2
Sevoflurane group	7.23 ± 0.12	7.83 ± 0.18*	8.53 ± 0.28*#
Propofol group	7.18 ± 0.11	8.18 ± 0.21*	9.35 ± 0.31*#
T	0.341	1.832	3.905
*p*	.786	.095	.000

*Indicates comparison with T0, *p < .05; ^#^Indicates comparison with T1, #*p* < .05.

### Comparison of clinical effects between the two groups

The effective rate of the propofol group was higher than that of the control group after 7 days, but the difference was not statistically significant (*p* > .05). There was no significant difference between the two groups (*p* > .05). See Table 3. The overall clinical effects at 7 days after the procedure was categorised as: *A. Significant effect:* fever, cough and other clinical symptoms completely disappeared, no abnormalities found on chest X-ray examination; *B. Effective:* fever, cough and other clinical symptoms were relieved. Chest X-ray examination showed that the lung infiltration was reduced by more than 50%. *C. invalid:* the clinical symptoms of the child and the chest X-ray images did not change significantly.

**Table 3.** Comparison of clinical effects between the two groups (*n* (%)).

Group	*N*	Significant effect	effective	invalid
Sevoflurane group	30	12	17	1
Propofol group	30	14	16	0
χ2		2.248	0.538	0.375
P		.138	.463	.752

The overall clinical effects at 7 days after the procedure was categorized as: *A. Significant effect:* fever, cough and other clinical symptoms completely disappeared, no abnormalities found on chest X-ray examination; *B. Effective:* fever, cough and other clinical symptoms were relieved. Chest X-ray examination showed that the lung infiltration was reduced by more than 50%. *C. invalid:* the clinical symptoms of the child and the chest X-ray images did not change significantly.

### Comparison of postoperative recovery in two groups of children

There was no significant difference in body temperature recovery, cough disappearance, arpeggio disappearance and hospitalization time between the propofol group and the sevoflurane group (*p* > .05), as shown in Table 4.

**Table 4.** Comparison of postoperative recovery in two groups of children ($\bar{x}\pm s,$ *d*).

Group	*N*	Body temperature recovery (days)	Cough disappears (days)	Lung sound clears (days)	Hospital stay (days)
Sevoflurane group	30	3.64 ± 1.05	5.85 ± 1.24	6.74 ± 1.45	7.94 ± 2.07
Propofol group	30	3.84 ± 0.94	5.21 ± 1.05	5.67 ± 1.09	7.36 ± 1.35
P		.603	.205	.056	.305

## Conclusion

In recent years, the incidence of mycoplasma pneumoniae pneumonia (MPP) in children has increased steadily and gradually. The pathogenesis of SMPP is still unclear, likely through alveolar infiltration with neutrophils and lymphocytes, lymphocyte/plasma cell infiltrates in the peri-bronchovascular area, and immune dysfunction. Most investigations found that the immunological mechanism plays a major role in the pathogenesis and development of SMPP [8,9]. The clinical manifestations of SMPP include high fever, cough, and decreased pulmonary function. It not only induces atypical pneumonia, but also may cause multiple organ damage, affecting the general health and potential loss of life in the paediatric population.

Fibreoptic bronchoscopy and alveolar lavage are important strategies for the treatment of SMPP. These procedures are usually be done under general anaesthesia. Propofol and sevoflurane are commonly used anaesthetic agents in children. Both have the characteristics of high anaesthetic efficacy, rapid induction and fast recovery [10]. Recent experimental and clinical studies showed that sevoflurane may have immunosuppressive effects, and multiple studies confirmed that the immune function of children with SMPP had been reduced [4,11]. It is very concerning because the use of sevoflurane and other anaesthetic agents may superimpose more immunosuppressive effects on the children with SMPP and these anaesthetics may further negatively affect the postoperative recovery and worsen the prognosis of these children [10]. However, the results of this study indicated that there was no significant difference between the two groups in terms of the overall clinical outcome 7 days after surgery. There was no significant difference between the two groups in fever occurrence, cough improvement, loss of voice, and length of hospital stay.

The immune system in human beings includes humoral immunity and cellular immunity. T cells are the main cells involved in cellular immunity. Helper T cells (CD4+) and cytotoxic T cells (CD8+) are the main subpopulations of T lymphocytes. The CD4+/CD8 + ratio can reflect the status of cellular immune function of the body. The decrease of CD3+ and CD4+/CD8 + ratios indicates that the immune function of the body is compromised. The reduction of cellular immune function will not only affect the recovery after surgery, but also predispose the patient to infection of other pathogens. The level of inflammation affects prognosis [12]. To further investigate the effects of sevoflurane and propofol on the immune function in the paediatric patient population, we examined and analysed the immune cells in peripheral blood in paediatric patents. Our results also showed that CD3+ and CD4+/CD8+ ratio were reduced to some extent in both groups at the end of anaesthesia, and the cell immunity was obviously restored in 24 h after anaesthesia. The CD3+, CD4+ and CD4+/CD8+ in the propofol group were significantly higher than those in the sevoflurane group. This might be because sevoflurane can cause the destruction of mitochondrial membrane potential, thereby promoting its release of cytochrome C, directly inhibiting the acquisition of lymphocyte ATP and promoting apoptosis of peripheral blood lymphocytes [13]. In addition, sevoflurane also inhibits the activation of human T lymphocyte activator protein-1 transcription factor, thereby interfering with the P38 MAPK cascade, resulting in a decrease in T cells in human peripheral blood [14]. Yuki et al. also found that isoflurane may inhibit lymphocyte function by inhibiting the binding of lymphocyte-associated factor LFA-1 to its ligand, suggesting that inhibition of lymphocyte function by inhaled anaesthetics is a combination of multiple pathways [15]. Song et al. believed that low concentrations of propofol do not cause lymphocyte apoptosis [16]. These studies have shown that clinical doses of propofol have little effect on lymphocyte function and seemed to indicate propofol is less immunosuppressive in children with SMPP undergoing bronchoscopy than sevoflurane, which is helpful for postoperative recovery.

In addition, CD4 + cells can be divided into Th1, Th2, Th9, Th17, Th22, Treg and Tfh cells [17] Th1/Th2 balance is the basis of immune regulation. Th1 cells can secrete cytokines such as IL-2, IFN-ꭇ and TNF-α, promote cell-mediated immune response and kill microbial pathogens intracellularly. Th2 cells can secrete cytokines such as IL-4, IL-6 and IL-10, which can promote humoral immunity, and attack extracellular microbial pathogens and secrete protective antibodies. Usually, Th1 and Th2 subsets of cells interact and restrict each other by secreting cytokines to maintain the balance of immunity. The imbalance of Th1/Th2 in the immune system can lead to clinical manifestations of allergic diseases. Studies have shown that allergic diseases and respiratory tract infections can down-regulate Th1 cytokines and/or up-regulate Th2 cytokines, thereby altering the balance status of Th1/Th2. After infection of the mycoplasma pneumonia pathogen, excessive humoral immune response in the host plays a crucial role in the development of SMPP. SMPP is closely related to the imbalance of Th1/Th2 ratio. Down-regulation of Th1 cytokines and/or up-regulation of Th2 cytokines lead Th2 cells to be dominant and thus the imbalance of cellular and humoral immunity [17]. Ji et al. found that propofol can up-regulate Th1/Th2 levels, which is consistent with the fact that propofol can maintain Th1/Th2 homeostasis better than sevoflurane in this study, because cytokines secreted by Th1 mainly activate cells involved in cellular immunity, and cytokines secreted by Th2 cells can stimulate the secretion of protective antibodies and promote humoral immunity, so propofol can promote Th1/Th2 homeostasis [18]. The increase of Th1/Th2 ratio is beneficial to maintain cellular immune function and to patients with compromised immune function. This may be related to the fact that propofol is more effective than sevoflurane in reducing surgical stress and plasma cortisol level. Finally, We do not believe the effects we observed are from remifentanil because #1 we used remifentanil in both groups with same dosing regimen; #2 The minor effect of remifentanil reported in the literature seems to be more related to humeral immune function and non-specific immune function.

The limitation of this study is that the sample size is insufficient and there is no follow-up investigation on the selected children, so the results of this study can not be applied to all patients, we still need to carry out a long-term large sample study.

In summary, this study found that propofol administered in children with SMPP had less inhibitory effect on T lymphocytes than inhalational sevoflurane, had little inhibitory effect on the differentiation of Th cells into Th1 cells, and better preserve Th1/Th2 ratio and maintain the balanced immune function.

## Data Availability

Data sharing is available by emailing the corresponding author.
